# Frequent Transmission of Gonorrhea in Men Who Have Sex with Men

**DOI:** 10.3201/eid2301.161205

**Published:** 2017-01

**Authors:** Christopher K. Fairley, Jane S. Hocking, Lei Zhang, Eric P.F. Chow

**Affiliations:** Melbourne Sexual Health Centre, Melbourne, Victoria, Australia (C.K. Fairley, J.S. Hocking, L. Zhang, E.P.F. Chow);; Monash University, Melbourne (C.K. Fairley, L. Zhang, E.P.F. Chow);; University of Melbourne, Melbourne (J.S. Hocking)

**Keywords:** *Neisseria gonorrhoeae*, male homosexuality, syphilis, men who have sex with men, MSM, transmission, kissing, bacteria, antimicrobial resistance, gonorrhea

## Abstract

The rate of gonorrhea is much higher in men who have sex with men than in heterosexuals. Because of unique behavioral characteristics, asymptomatic sites of infection, mainly the pharynx, are principal drivers of gonorrhea prevalence in men who have sex with men. On the basis of this observation, we call for interventions.

The rates of sexually transmitted infections are rising rapidly in men who have sex with men (MSM) (*1*). Gonorrhea is of particular concern because rising rates will increase the probability of antimicrobial drug resistance (*2*). In response, the Centers for Disease Control and Prevention has recommended reducing the prevalence of gonorrhea as a key strategy to mitigate against antimicriobial resistance (*2*). However, reducing prevalence requires understanding why gonorrhea is so common in MSM. We suggest that specific sexual practices of MSM result in them having a high prevalence of asymptomatic infection in particular anatomic sites and that these infections are the primary drivers of transmission (*3*).

In heterosexuals, the primary sites of gonorrheal infection are the urethra in men and cervix in women (*4*). Most heterosexual men with urethral infection become symptomatic and quickly seek healthcare (after a few days) (*5*). About half of women are asymptomatic, and thus they take longer to seek healthcare than men (*5*,*6*).

In MSM, 3 sites are commonly infected: pharynx, rectum, and urethra (*7*). In a Seattle clinic, the proportion of MSM with pharyngeal gonorrhea was 6.5%, rectal gonorrhea 9.7%, and urethral gonorrhea 5.5% (*7*). Almost all urethral infections were symptomatic (96%), but most pharyngeal and rectal infections were asymptomatic. Most pharyngeal or rectal infections (58%) were not associated with urethral infection (*7*).

An additional factor favoring the persistence of gonorrhea-infected sites in MSM is their lower rate of partner notification compared with heterosexuals (*8*). This behavior creates a scenario in which men with pharyngeal or rectal gonorrhea often go untreated, even if they transmit an infection to the urethra of a sex partner. This longer duration of infectiousness translates into a higher reproductive rate for gonorrhea in MSM compared with heterosexuals, independent of the number of sexual partners. Determining the key drivers of the reproductive rate for gonorrhea in MSM involves characterizing transmission between anatomic sites, which requires quantifying the site-specific sexual practices of MSM. Studies assessing the most recent sexual acts among MSM show that most have kissed (75%), practiced mutual masturbation (64%), or had oral sex (77%) (*9*); oro-anal sex (25%) and penile-anal sex (35%) are less common (*9*). In contrast, in heterosexuals, penile-vaginal sex occurs in 95% of most recent sexual acts; therefore, most sexual acts between heterosexuals in which gonorrhea transmission occurs will lead to symptomatic infections that prompt them to seek treatment (*9*,*10*).

One behavior that may be important for transmitting gonorrhea that has not been well studied is kissing (*11*). Kissing has not been asked about in any national sex surveys and only occasionally in clinical sexually transmitted infection studies (*9*). We were unable to find any published studies on kissing partners in which sex did not occur (termed kissing-only partners) either in heterosexuals or MSM, besides the data we recently presented (*3*). We surveyed 1,151 MSM attending our clinic in 2016 and found a mean of 3.7 kissing-only partners and a mean of 4.5 kissing and sex partners in the previous 3 months (*3*) (Technical Appendix Figure 1). Kissing-only partners were much more common among younger MSM, who are at substantially higher risk for gonorrhea than older MSM (*3*,*12*). The reason for this preponderance of gonorrhea in young MSM is currently unknown but is consistent with and could be explained by kissing being an important transmission route.

We determined what we consider to be the accepted transmission routes for gonorrhea by anatomic site in MSM (Figure, panel A), although one should acknowledge that no studies have reported site-specific gonorrhea transmission between MSM partners. Major textbooks and published studies indicate the penis is key to gonorrhea transmission between men (Figure, panel A) (*4*). Studies suggest that urethral infection is largely acquired from unprotected anal sex, with perhaps one third of cases acquired by receiving oral sex (Technical Appendix Table).

**Figure:**
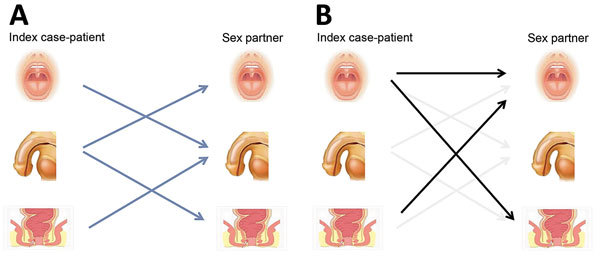
Traditional and proposed transmission models for gonorrhea in men who have sex with men (MSM). A) Generally accepted transmission routes (arrows) for gonorrhea between sites in MSM from an infected index case-patient to an uninfected sexual partner. B) Additional proposed transmission routes (dark arrows) compared with accepted transmission routes (light arrows). MSM, men who have sex with men.

Relatively little research has been done on gonorrhea transmission not involving the penis. Some observational studies support the potential transmission of gonorrhea between the pharynx and rectum, although this is not consistently described as a route of transmission in major textbooks (*4*). Studies have shown that receptive oro-anal sex has been associated with rectal infection, and oro-anal sex has been associated with pharyngeal gonorrhea (Technical Appendix Table).

We propose new models of gonorrhea transmission: throat-to-throat transmission through kissing and throat-to-anus transmission (and vice versa) through oro-anal sex (Figure, panel B). We propose that transmission to the penis occurs but contributes little to the reproductive rate because it is present there a short time relative to the other anatomic sites.

Unfortunately, there are few studies on gonorrhea transmission between the throats of sex partners to support or refute our suggestion. We did, however, find case reports of transmission through kissing from >40 years ago, and kissing is a well-recognized transmission route for other *Neisseria* species (*11*,*13*). In a matched, case–control study of 15- to 19-year-olds, intimate kissing with multiple partners was associated with an odds ratio of 3.7 for meningococcal disease (*13*). One of the few cohort studies in MSM to ask about kissing showed it to be significantly associated with pharyngeal gonorrhea (Technical Appendix Table), but few studies have examined kissing behavior.

The frequent detection of gonorrhea in the saliva of men with pharyngeal infection suggests saliva likely plays a role in gonorrhea transmission (*14*). Saliva is central to oral sex, oro-anal sex, and even penile-anal sex; saliva is commonly used as lubricant (*14*).

Transmission models for gonorrhea in MSM should be consistent with current site-specific prevalence and incidence. We estimated prevalence and incidence of pharyngeal and anal gonorrhea from 3,034 MSM attending a Seattle clinic on the basis of site-specific duration data (Technical Appendix Figure 2) (*7*,*15*). The incidence of urethral gonorrhea was ≈5.5/100 person-years, and we estimated the prevalence among MSM to be low (0.24%) because the infections are often of short duration due to their treatable and symptomatic nature. It is difficult to see how, even with frequent changes in sex partners, the estimated incidence of pharyngeal infection (26/100 person-years) could arise from urethral infection, given its low prevalence.

There are several implications if our model of transmission is correct. First, a preventive approach using condoms will not work because, unlike heterosexuals, the penis is not responsible for most gonorrhea transmission among MSM. Second, the screening that is advocated annually for MSM would need to be much more frequent to reduce the disease reproductive rate. MSM taking pre-exposure prophylaxis for HIV are screened every 3 months; this screening frequency might be sufficient to reduce gonorrhea prevalence. Third, our model suggests reducing pharyngeal duration and transmissibility is needed for gonorrhea control, and we call for suggestions of interventions that might achieve this. One approach we are investigating is an antibacterial mouthwash (clinical trial no. ACTRN12616000247471), following up some of our earlier data.

Finally, it is possible that the rapidly rising rates of syphilis in MSM may share similarities with gonorrhea transmission. Syphilis is also uncommon in heterosexuals and more likely to be asymptomatic in MSM with anal infection. When interventions are being tested for their effects on gonorrhea transmission, investigators might consider including syphilis as an outcome.

Technical Appendix Evidence suggesting that kissing contributes to gonorrhea transmission among men who have sex with men.
