# Supplementary material for: Frequent Transmission of Gonorrhea in Men Who Have Sex with Men
Source: Emerg Infect Dis. 2017 Jan;23(1):102–4. doi: 10.3201/eid2301.161205 (PMC5176237; doi:10.3201/eid2301.161205)
Supplement: Technical Appendix — Evidence suggesting that kissing contributes to gonorrhea transmission among men who have sex with men. [file 16-1205-Techapp-s1.pdf]

Article DOI: http://dx.doi.org/10.3201/eid2301.161205

Frequent Transmission of Gonorrhea in Men Who Have Sex with Men

Technical Appendix

Technical Appendix Table. References referring to the throat as a transmission route for gonorrhea between men*

Transmission route	Comment	References
Throat-to-penis and anus-to-penis	Studies demonstrate that ≈1/3 of urethral gonorrhea in MSM is acquired from oral sex and ≈2/3 from anal sex.	(*1*–*3*)
Throat-to-anus and anus-to-throat	A risk factor for anal gonorrhea was receiving oro-anal sex. Incidence of pharyngeal gonorrhea was higher in those who reported insertive oro-anal sex. Saliva is culture positive in a substantial proportion of throat-positive cases.	(*3*–*7*)
Throat-to-throat	The incidence of pharyngeal gonorrhea was higher in men who reported tongue kissing. A case report in which kissing was the most likely explanation for transmission.	(*4*,*8*)

*MSM, men who have sex with men.

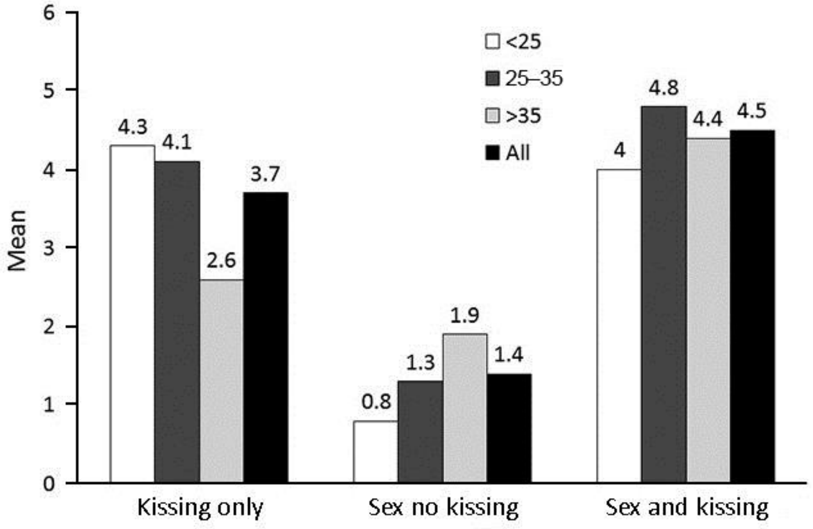

Technical Appendix Figure 1. Kissing and sex partners among men who have sex with men (MSM) by age in previous 3 months. The results from a survey of 1,151 MSM attending Melbourne Sexual Health Centre in 2016. Kissing only refers to the mean number of tongue kissing partners without any sex (oral or anal). Sex no kissing refers to the mean number of sex partners in which there was no tongue kissing. Sex and kissing refers to the mean number of sex partners in which tongue kissing occurred.

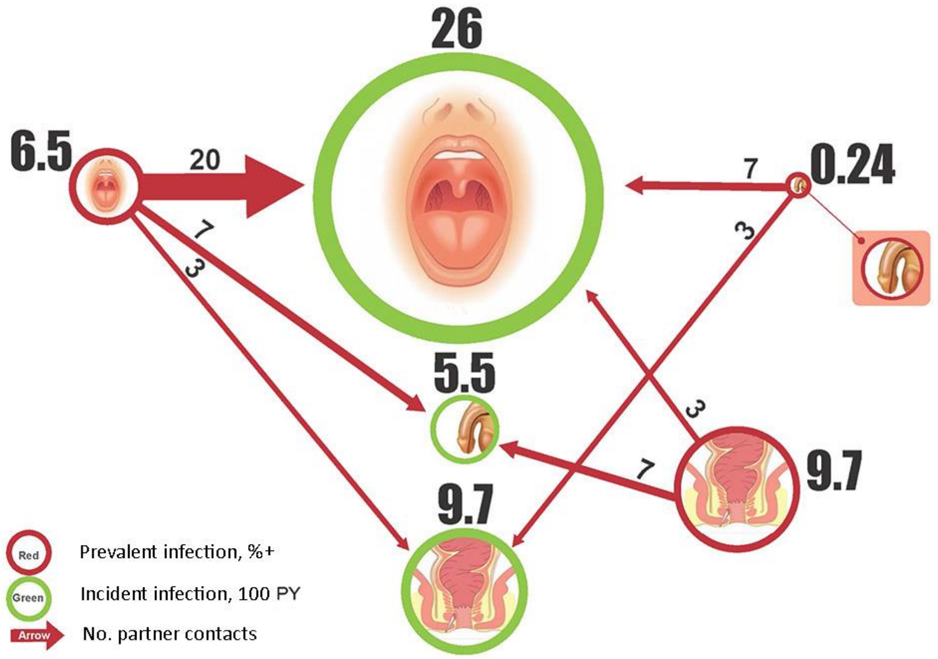

Technical Appendix Figure 2. The prevalence and incidence of gonorrhea and sexual practices in men who have sex with men (MSM) by anatomic site. Prevalence data of pharyngeal and rectal gonorrhea and annual incidence of urethral gonorrhea from Barbee et al. (*9*). We estimated the incidence of pharyngeal and rectal gonorrhea and prevalence of urethral gonorrhea from various references (*9*–*11*). Red circles show the prevalence of gonorrhea at specific sites of potential sex partners. Green circles show site-specific incidence. The size of the circles is proportional to the prevalence (proportion positive) and incidence (per 100 person-years [PY]). The line thickness and numbers on the lines refer to the number of partners with this sexual exposure in a given year, considering an average of 10 sex partners a year (*12*). Our data on kissing-only partners indicates that it is about double the number of sex partners (i.e., 20 persons) (*13*). Prevalence of urethral gonorrhea was calculated by taking the incidence of 5.5 and assuming it was infectious for 1 week (5.5/52) and assuming 10% were asymptomatic and persisted for 3 months untreated ([5.5*0.1]/4). Penis-to-penis and anus-to-anus transmission were assumed to be rare.

References

1. Barbee LA, Khosropour CM, Dombrowski JC, Manhart LE, Golden MR. An estimate of the proportion of symptomatic gonococcal, chlamydial and non-gonococcal non-chlamydial urethritis attributable to oral sex among men who have sex with men: a case-control study. Sex Transm Infect. 2016;92:155–60. PubMed http://dx.doi.org/10.1136/sextrans-2015-052214

2. Nash JL, Hocking JS, Read TR, Chen MY, Bradshaw CS, Forcey DS, et al. Contribution of sexual practices (other than anal sex) to bacterial sexually transmitted infection transmission in men who have sex with men: a cross-sectional analysis using electronic health records. Sex Transm Infect. 2014;90:55–7. PubMed http://dx.doi.org/10.1136/sextrans-2013-051103

3. Jin F, Prestage GP, Mao L, Kippax SC, Pell CM, Donovan B, et al. Incidence and risk factors for urethral and anal gonorrhoea and chlamydia in a cohort of HIV-negative homosexual men: the Health in Men Study. Sex Transm Infect. 2007;83:113–9. PubMed http://dx.doi.org/10.1136/sti.2006.021915

4. Templeton DJ, Jin F, McNally LP, Imrie JC, Prestage GP, Donovan B, et al. Prevalence, incidence and risk factors for pharyngeal gonorrhoea in a community-based HIV-negative cohort of homosexual men in Sydney, Australia. Sex Transm Infect. 2010;86:90–6. PubMed http://dx.doi.org/10.1136/sti.2009.036814

5. Chow EP, Lee D, Tabrizi SN, Phillips S, Snow A, Cook S, et al. Detection of *Neisseria gonorrhoeae* in the pharynx and saliva: implications for gonorrhoea transmission. Sex Transm Infect. 2016;92:347–9. PubMed http://dx.doi.org/10.1136/sextrans-2015-052399

6. Hutt DM, Judson FN. Epidemiology and treatment of oropharyngeal gonorrhea. Ann Intern Med. 1986;104:655–8. PubMed http://dx.doi.org/10.7326/0003-4819-104-5-655

7. Hallqvist L, Lindgren S. Gonorrhoea of the throat at a venereological clinic. Incidence and results of treatment. Br J Vener Dis. 1975;51:395–7. PubMed

8. Willmott FE. Transfer of gonococcal pharyngitis by kissing? Br J Vener Dis. 1974;50:317–8. PubMed

9. Barbee LA, Dombrowski JC, Kerani R, Golden MR. Effect of nucleic acid amplification testing on detection of extragenital gonorrhea and chlamydial infections in men who have sex with men sexually transmitted disease clinic patients. Sex Transm Dis. 2014;41:168–72. PubMed http://dx.doi.org/10.1097/OLQ.0000000000000093

10. Garnett GP, Mertz KJ, Finelli L, Levine WC, St Louis ME. The transmission dynamics of gonorrhoea: modelling the reported behaviour of infected patients from Newark, New Jersey. Philos Trans R Soc Lond B Biol Sci. 1999;354:787–97. PubMed http://dx.doi.org/10.1098/rstb.1999.0431

11. Chow EP, Camilleri S, Ward C, Huffam S, Chen MY, Bradshaw CS, et al. Duration of gonorrhoea and chlamydia infection at the pharynx and rectum among men who have sex with men: a systematic review. Sex Health. 2016;13:199–204. PubMed

12. Rosenberger JG, Reece M, Schick V, Herbenick D, Novak DS, Van Der Pol B, et al. Sexual behaviors and situational characteristics of most recent male-partnered sexual event among gay and bisexually identified men in the United States. J Sex Med. 2011;8:3040–50. PubMed http://dx.doi.org/10.1111/j.1743-6109.2011.02438.x

13. Fairley CK. Oropharyngeal transmission of gonorrhoea—is it the key? Presented at: British Association for Sexual Health and HIV Annual Conference; Oxford, UK; 2016 Jul 10–12.
